# Are screening methods useful in feature selection? An empirical study

**DOI:** 10.1371/journal.pone.0220842

**Published:** 2019-09-11

**Authors:** Mingyuan Wang, Adrian Barbu

**Affiliations:** Statistics Department, Florida State University, Tallahassee, Florida, United States of America; Dartmouth College Geisel School of Medicine, UNITED STATES

## Abstract

Filter or screening methods are often used as a preprocessing step for reducing the number of variables used by a learning algorithm in obtaining a classification or regression model. While there are many such filter methods, there is a need for an objective evaluation of these methods. Such an evaluation is needed to compare them with each other and also to answer whether they are at all useful, or a learning algorithm could do a better job without them. For this purpose, many popular screening methods are partnered in this paper with three regression learners and five classification learners and evaluated on ten real datasets to obtain accuracy criteria such as R-square and area under the ROC curve (AUC). The obtained results are compared through curve plots and comparison tables in order to find out whether screening methods help improve the performance of learning algorithms and how they fare with each other. Our findings revealed that the screening methods were useful in improving the prediction of the best learner on two regression and two classification datasets out of the ten datasets evaluated.

## Introduction

For the past few decades, with the rapid development of online social platforms and information collection technology, the concept of big data grew from a novel terminology in the past to one of the most powerful resources in present day. Especially in recent years, the sample sizes and feature space dimensions of datasets rose to levels beyond precedent. This development poses great challenges for machine learning in extracting the relevant variables and in building accurate predictive models on such large datasets.

One of the most popular machine learning tasks is feature selection, which consists of extracting meaningful features (variables) from the data, with the goal of obtaining better prediction on unseen data, or obtaining better insight on the underlying mechanisms driving the response.

Feature selection methods have grown into a large family nowadays, with T-score [1], Mutual Information [2], Relief [3], Lasso [4], and MRMR [5] as some of the more popular examples.

There are three categories of feature selection methods: screening methods (a.k.a. filter methods), wrapper methods and embedded methods. Screening methods are independent of the model learned. This makes them less computational complex. However for the same reason, screening methods tend to ignore more complex feature traits. They generally provide the lowest improvement among the three. Wrapper methods use a learning algorithm (learner) to evaluate feature importance, which often leads to a better performance. But good performance comes with the possibility of overfitting, and a much higher computational demand. Embedded methods combine the feature selection and the model learning together, which generally makes them faster than the wrapper methods. However, the way that the feature selection and the learning are combined together makes them specific to the learning algorithm used. The features selected by one kind of learner-specific embedded method may not useful for other kinds of learners. In this study, we focus our attention on screening methods and would like an unbiased answer to the following questions:

- Do screening methods help to build good predictive models, or comparable models can be obtained without them?
- How do the existing screening methods compare with each other in terms of predictive capabilities, which one is the best and which one is the worst?

To answer these questions, we evaluated different screening methods (three for regression and seven for classification) on ten real datasets, five for regression and five for classification. The screening methods and the datasets will be described in the Methods section, but here we present our main findings.

The screening methods themselves cannot provide predictive models. For that purpose, different supervised learning algorithms such as SVM, Feature Selection with Annealing (FSA), Boosted Trees, and Naive Bayes were employed to construct the predictive models on the features selected by the screening methods.

For both regression and classification, experiments indicate that the screening methods are sometimes useful, in the sense they help obtaining better predictive models on some datasets. The findings are summarized in the comparison tables from the Results section.

Through our comparison study, we intend to provide researchers with a clear understanding of some of the well known screening (filter) methods and their performance of handling high-dimensional real data.

### Related work

The focus of this study is to examine the effect of screening (filter) methods on obtaining good predictive models on high-dimensional datasets. The recent literature contains several works that compare feature selection methods, including screening methods.

A recent feature selection survey [6] from Arizona State University (ASU) shows a comprehensive feature selection contents, studying feature selection methods from different data type perspectives. The survey is very broad, examining both supervised and unsupervised learning using binary and multi-class data, whereas our study focuses on supervised learning on regression and binary classification problems. The ASU study evaluates many classification datasets, but it does not have our goal of comparing feature screening methods and testing whether they are useful in practice or not. In this respect, we found some issues with the ASU study and we corrected them in this paper. First, the ASU study uses the misclassification error as a measure of the predictive capability of a classifier. The misclassification error is sensitive to the choice of threshold, and is a more noisy measure than the AUC (area under the ROC curve). In our work we used the AUC instead, and obtained performance curves that have less noise, as it will be seen in experiments. Second, the ASU study obtains the results with 10-fold cross-validation, and are not averaged over multiple independent runs. In our work we used 7 independent runs of 7-fold cross-validation to further increase the power of our statistical tests. Third, we draw our comparisons and conclusions using statistical methods based on paired *t*-tests to obtain groups of similarly performing methods. An earlier version of the ASU report is [7], which is an overview of different types of feature selection methods for classification.

In [8] are evaluated feature selection methods for flat features including filter methods, wrapper methods and embedded methods. However, tests are only conducted on low-dimensional datasets. In contrast we evaluate the filter methods on high dimensional datasets with 500-20,000 features and in many instances with more features than observations. Moreover, our goal is to compare filter methods themselves, not the filter-learning algorithm combination, since different datasets could have different algorithms that are appropriate (e.g. linear vs nonlinear). We achieve this goal by employing many learning algorithms and choosing the best one for each filter method and each dataset.

A comprehensive overview of the feature selection work done in recent years is shown in [9]. It covers feature selection methods including filter, wrapper, embedded and hybrid methods as well as structured and streaming feature selection. The article also discusses existing application of these feature selection methods in different fields such as text mining, image processing, industry and bioinformatics.

Recently [10] gave another detailed and broad overview of feature selection methods. The authors conducted their studies of many categories of feature selection methods, including but not limited to supervised, unsupervised, semi-supervised, online learning and deep learning. An experiment involving five feature selection methods was conducted on classification data. All five methods are either filter or wrapper methods. However they conducted their experiments on only two datasets, and the didn’t consider the performance of the learning algorithms without any feature selection as a comparison baseline. Therefore the paper fails to show how much the feature selection methods could improve accuracy or whether they improved accuracy at all.

From a very interesting and unique standing point, [11] is an overview that focuses on the challenges currently facing feature selection research. They propose some solutions while at the same time reviewing existing feature selection methods. In [12] is evaluated the existing Relief method and some of its variants. The authors implemented and expanded these methods in an open source framework called ReBATE (Relief-Based Algorithm Training Environment). They described these methods in great detail and conducted simulation experiments with prior knowledge of the true features. They used a very vast simulated data pool with many varieties. The Relief variants were also compared with three other filter methods, using as performance measure the rate of detection of the true features. However, the paper didn’t show if these methods can improve the performance of machine learning algorithms or if the improvement persists on real data.

Two other studies of feature selection methods are [13] and [14]. In contrast to our study, they solely focus on unsupervised learning.

With the development of feature selection research, some well written feature selection software frameworks were also introduced. FeatureSelect [15] is a newly introduced such framework, which evaluated multiple trending feature selection methods on eight real datasets. Results were compared using various statistical measures such as accuracy, precision, false positive rate and sensitivity. Their studies also evaluated five filter methods. Because the experiment didn’t have learning algorithms without feature selection method as a benchmark, it again fails to show if using feature selection methods is better than not using them on these datasets. IFeature [16] is another feature selection software framework dedicated to Python.

Some earlier studies also exist in this field (Guyon and Elisseeff, 2003 [17], Sanchez-Marono et al.,2007 [18], Saeys et al.,2007 [19]).

## Methods

Experiments were conducted separately for regression and classification. For regression, the screening methods were Correlation, Mutual Information [2], and RReliefF [20]. These screening methods were combined with learners including Feature Selection with Annealing (FSA) [21], Ridge Regression, and Boosted Regression Trees.

For classification, the screening methods were T-score [1], Mutual Information [2], Relief [3], Minimum Redundancy Maximum Relevance (MRMR) [5], Chi-square score [22], Fisher score [23], and Gini index [24]. They were combined with learners including FSA [21], Logistic Regression, Naive Bayes, SVM, and Boosted Decision Trees.

Among these screening methods, Mutual information, Correlation, Gini index, Fisher-score, Chi-square score and T-score select features individually. In contrast, MRMR requires to calculate the redundancy between the already selected features and the current feature, and Relief requires to calculate the distance between two observations using the Euclidean norm, so that one can determine the nearest neighbor with the same label and with a different label. The calculation of the Euclidean norm involves all the feature values. Consequently, these two methods select features in combination and are slower than the other methods.

### Evaluation of screening methods

The predictors of all datasets were normalized to zero mean and standard deviation 1 in a pre-processing step. For each dataset, experimental results were obtained as the average of 7 independent runs of 7-fold cross-validation. For each run, a random permutation of the dataset was generated and the data was split into seven approximately equal subsets according to the permutation. Then a standard full 7-fold cross-validation was performed as follows. Each fold consists of testing on one of the subsets after training on the other six. This procedure was run with each of the seven subsets as the test set and the other six as the training set. For each fold, each one of the screening methods mentioned above was used to reduce the dimension of the feature space to the desired size, then a learning algorithm using preset parameter values was applied on the selected features to obtain the model. The predictions of the model on the test subset for each fold were combined to obtain a vector of test predictions on the entire dataset, which was used to obtain performance measures (*R*^2^ for regression and AUC (Area under the ROC curve) for classification). To increase accuracy, these performance measures were averaged over seven independent runs on different permutations of the data.

To insure the consistency of the comparison, the number of features that were selected by each screening method was kept the same for each dataset. For each dataset (except Wikiface), 30 different values of the number of selected features were assigned. Plots were used to compare the average performance over the 7 cross-validated runs of different combinations of screening method and learner. Also for each combination, the optimal number of selected features was selected based on the maximum average test performance over the 7 cross-validated runs. Pairwise t-tests at the significance level *α* = 0.05 were used to compare between different combinations to see if they are significantly different.

### Construction of the tables of groups

Groups of screening method-learner combinations that are not significantly different from each other were constructed as follows (we use paired *t*-tests to obtain *p*-values when comparing different methods combinations and set 0.05 as the significance level in our experiment). The screening method-learner combinations are first sorted in descending order of their peak performance. Then starting from the first combination F downward, the last combination in the sequence that is not statistically significantly different from combination F is marked as combination L. All combinations between F and L are put into the same group. The same procedure was used for other combinations along the sequence. All these tables of groups are provided in the Supporting Information.

### Construction of the comparison tables

Comparison tables were established based on how many times each screening method-learner combination appeared in the group tables. Three kinds of counting methods were applied.

1. *The number of datasets where the screening method performed significantly better than no screening* for different learning algorithms. For each learning algorithm, it is the number of datasets on which the screening method appeared in higher group tiers than the same learning algorithm without screening.
2. *The number of datasets where the screening method was significantly better than the best performing algorithm with no screening (usefulness per dataset)*. For each dataset, we checked for each screening method whether it appeared with a learning algorithm in a higher group tier than the best learning algorithm without screening. The column named “Total Count” is generated for each screening method from the sum of the counts across all datasets.
3. *The number of datasets where each filter-learning algorithm combination was in the top performing group (top performing)*.

## Results

### Data sets

Five datasets were used for regression and five datasets for classification, with the specific dataset details given in Table 1.

**Table 1:** The datasets used for evaluating the screening methods. The parameter *τ* controls the number of selected features as $\left[{\left(4t\right)}^{\tau }\right],t=\bar{1,30}$.

Dataset	Learning type	Feature type	Number of features	Number of observations	*τ*
Mouse BMI [25]	Regression	Continuous	21575	294	1.825
Tumor [26]	Regression	Continuous	16790	1750	1.825
Indoorloc [27]	Regression	Continuous	520	20294	1.25
Wikiface [28]	Regression	Continuous	4096	53040	1.65
CoEPrA2006 [29]	Regression	Continuous	5787	133	1.68
Gisette [30]	Binary Classification	Continuous	5000	7000	1.73
Dexter [30]	Binary Classification	Continuous	20000	600	1.78
Madelon [30]	Binary Classification	Continuous	500	2600	1.25
SMK_CAN_187 [31]	Binary Classification	Continuous	19993	187	1.78
GLI_85 [32]	Binary Classification	Continuous	22283	85	1.78

The regression dataset Indoorloc is available on the UCI Machine Learning Repository [33]. The original dataset has eight indicator columns including longitude, latitude and so on. In our study, we only used the latitude as response. We combined the training and validation data files and deleted all duplicated observations due to the removing of the other seven indicator columns. The dataset Tumor was extracted from TCGA (The Cancer Genome Atlas). The response of this dataset is the survival time(in days) of the patient, and the predictors represent gene expression levels. The classification datasets Gisette, Dexter, Madelon are part of the NIPS 2003 Feature selection challenge [30] and are also available on the UCI Machine Learning Repository.

The dataset Wikiface is a regression problem of predicting the age of a person based on the person’s face image, and was obtained from the Wikiface images [28]. A CNN (Convolutional Neural Network) vgg-face [34] pre-trained for face recognition was applied to each face and the output of the 34-th layer was used to generate a 4096 feature vector for each face. This 4096 dimensional vector was used as the feature vector for age regression, with the age value from the original Wikiface data as the response.

### Regression results

The following results are based on the output generated using Matlab 2016b [35]. For RReliefF, correlation score, ridge regression and boosted regression trees we used their Matlab 2016b implementation. Mutual information for regression was implemented by ourselves. For FSA we used the Github implementation from its original authors [36].

#### Performance plots

**Fig 1:**
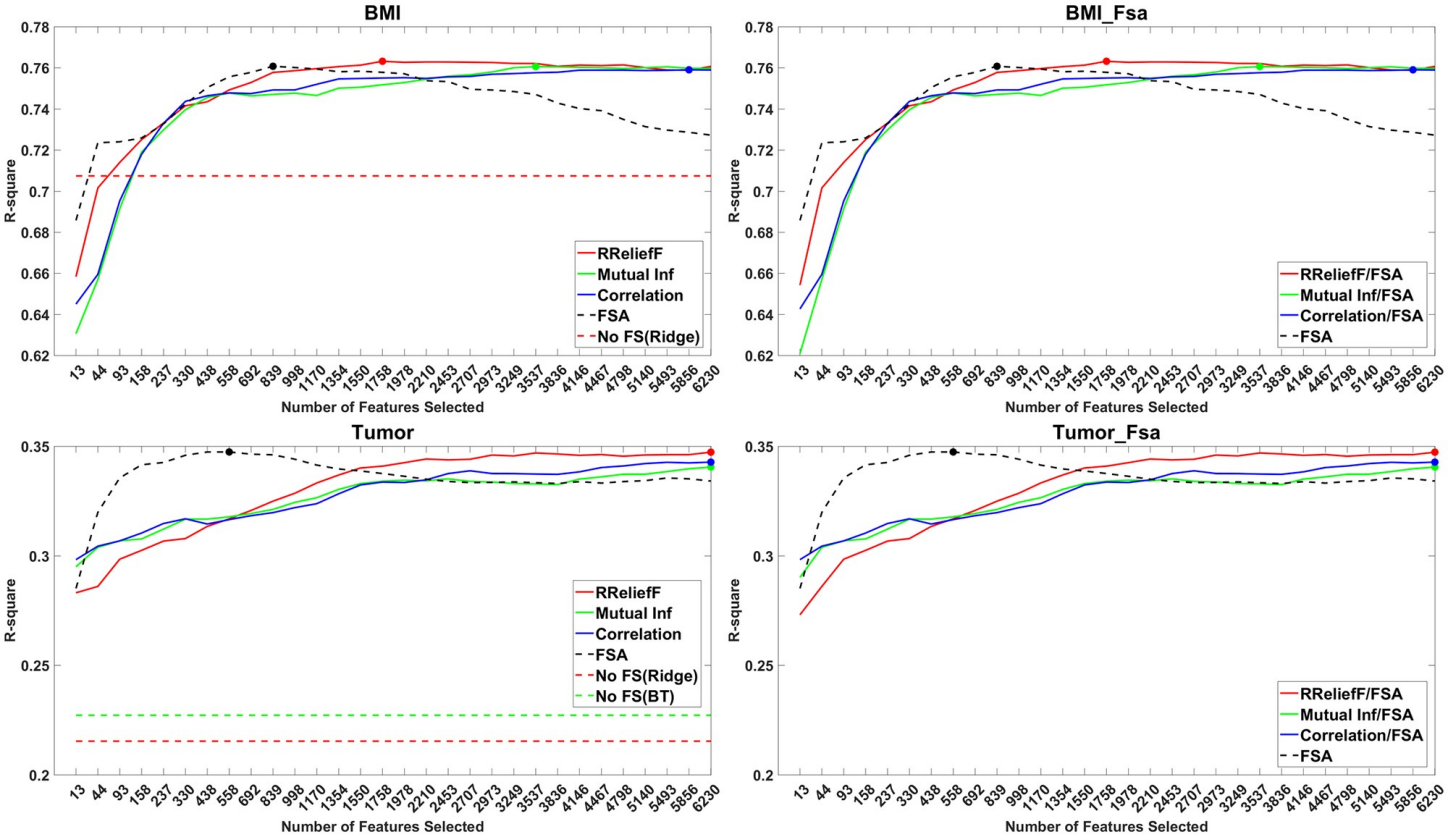
Performance plots of methods with and without feature screening. Left: for each screening method are shown the maximum *R*^2^ value across all learners. Right: *R*^2^ of the screening methods with the best learner for each dataset.

**Fig 2:**
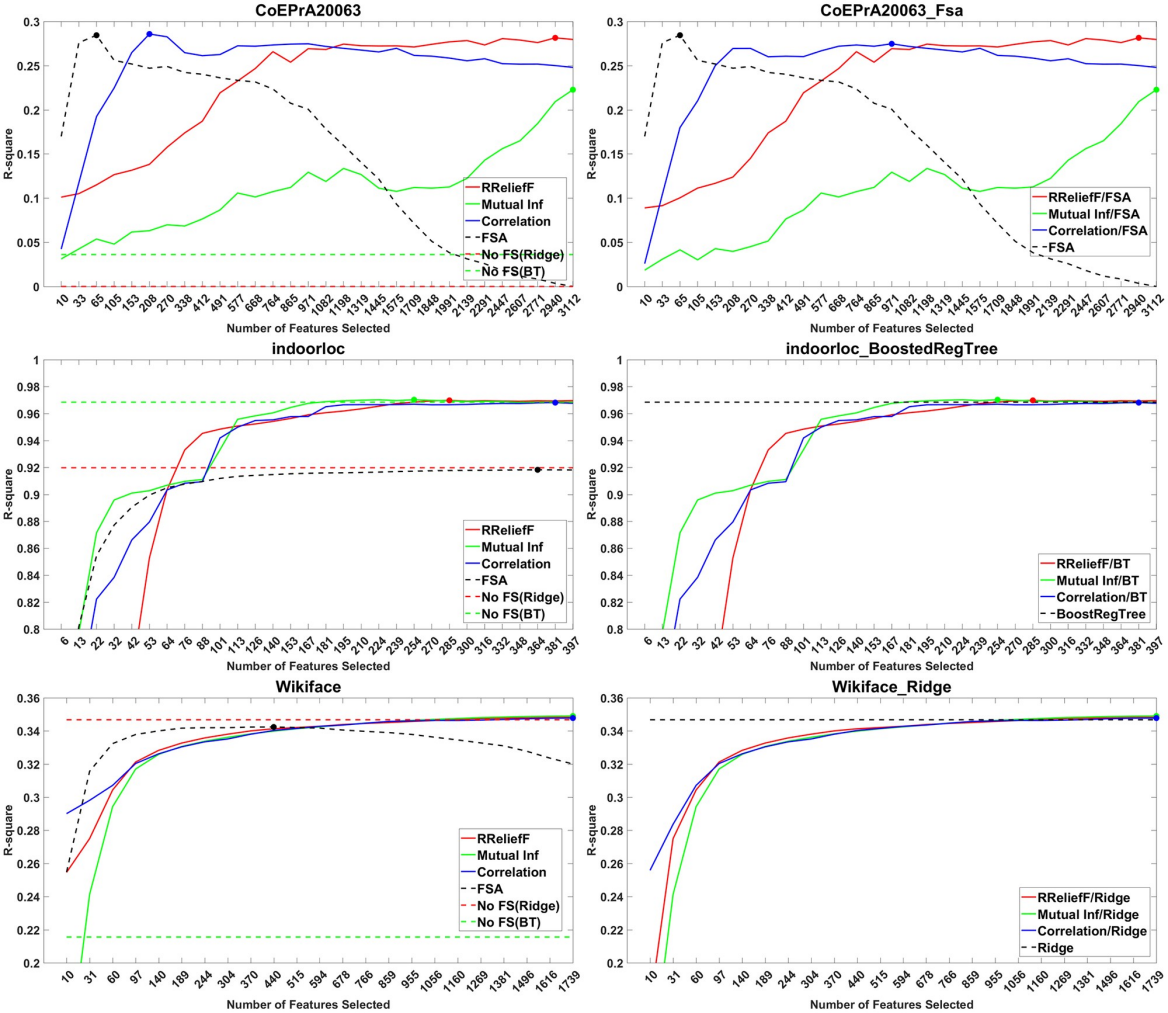
Performance plots of methods with and without feature screening. Left: for each screening method are shown the maximum *R*^2^ value across all learners. Right: *R*^2^ of the screening methods with the best learner for each dataset.

For the regression datasets, the plots from Figs 1 and 2 show the *R*^2^ value vs. the number *M*_*i*_ of selected features, where *M*_*i*_ = [(4*i*)^*τ*^], *i* = 1, …, 30. The value of *τ* for each dataset is given in Table 1.

In Fig 1, left, are shown the *R*^2^ of the best learning algorithm vs. the number of features selected by a screening method for the BMI and tumor datasets. Observe that these datasets are both gene expression datasets with many features and few observations. In Fig 1, right, are shown the *R*^2^ of FSA (the best overall learning algorithm) vs. the number of features selected by a screening method. Except a slightly higher value given by RReliefF on the BMI data, overall the screening methods did not show higher scores than that of the optimal regression learners for the BMI and Tumor datasets. The plots on the right show that the screening methods even needed to select more features to obtain similar performance to FSA without screening.

In Fig 2, left, are shown the *R*^2^ of the best learning algorithm vs. the number of features selected by a screening method for the other three regression datasets. In Fig 2, right, are shown the *R*^2^ of the best overall learning algorithm in each case (ridge for CoEPrA and Wikiface, boosted trees for Indoorloc) vs. the number of features selected by a screening method. From the plots we observe that screening methods give slightly better results than the learning algorithms without screening on the Indoorloc and Wikiface datasets. The statistical significance of the improvement can be seen in the table of groups from the supporting information or in the comparison tables below.

#### Comparison tables

The counts in the comparison table are based on the table of groups from the supporting information.

In Table 2 is shown the number of datasets where a filter method helps an algorithm perform significantly better, and the number of datasets where a screening method significantly improves a learning algorithm compared to the best performing learning algorithm without screening. It is shown that screening methods have relatively good performance with ridge regression and boosted regression trees on datasets tested. They work on 3-4 out of 5 datasets. RReliefF method and Mutual information method have slightly better performance than Correlation method when only comparing with the best learner without screening methods.

**Table 2:** Overview of the number of datasets where each feature screening method performed significantly better than no screening for different learning algorithms and than the best performing algorithm (larger numbers are better).

Screening Method	FSA	Ridge	Boost Tree	Best algorithm
RReliefF [20]	0	4	3	2
Mutual Information [2]	0	3	3	2
Correlation	0	4	3	1

In Table 3 is shown that Mutual Information and RReliefF worked on the Indoorloc dataset and all three screening methods worked on the Wikiface data. However, the screening methods didn’t provide performance improvement on the other three regression datasets. It is also shown that only in few occasions that screening methods harm the best learning algorithm. This is shown by the blank cells in table.

**Table 3:** Datasets on which a screening method performs similarly (shown by an “=” sign) or helps perform significantly better (shown by a “*” sign) than the best performing algorithm with no screening, and the total count for the “*” (larger numbers are better).

Screening Method	BMI	Tumor	CoEPrA	Indoorloc	Wikiface	Total Count
RReliefF	=	=	=	*	*	2
Mutual Information.	=			*	*	2
Correlation	=		=	=	*	1

In Table 4 is shown the counts of screening method-learner combinations that are in the top group. The combination of FSA with screening methods worked on more regression datasets than the other screening-learner combinations. However it was not a significant improvement compared to FSA without screening, which also worked on 3 out of 5 datasets.

**Table 4:** Number of datasets each combination was in the top performing group.

Learners	FSA	Ridge	Boost Tree
Filter			
RReliefF	3	0	1
Mutual Information	1	1	1
Correlation	2	1	0
—	3	0	0

In Table 5 is shown the number of times each screening method was in the top performing group. In the first column, these methods were counted together with the learning algorithms they were applied. So there can be at most 15 counts (For each screening method there are three learning algorithms and five datasets total) in each cells. The second column shows the counts withe the best learning algorithm for each method, so there can be at most 5 counts in each cell. The table shows that RReliefF has the best performance, which is larger that the worst performance (correlation) by 2. Among the three screening methods only RReliefF has a higher count than non screening.

**Table 5:** Ranking of feature screening methods for regression by the number of times each was in the top performing group. (larger numbers are better).

Screening Method	Top performing	
	Method-Algorithm	Method
RReliefF	4	4
Mutual Information	3	3
Correlation	3	2
No Screening	3	3

### Classification results

The following results are based on the output generated by Matlab 2016b. For the methods Relief, T-score, chi-square score, logistic regression, naive Bayes, SVM, boosted decision trees we used their Matlab 2016b implementation. For MRMR, Fisher score, and Gini index we used the ASU repository implementation [37]. Mutual information for classification was implemented by ourselves. Some of the implementations only accept discrete predictors, so the quantile-based discretization method [38] was used.

#### Performance plots

**Fig 3:**
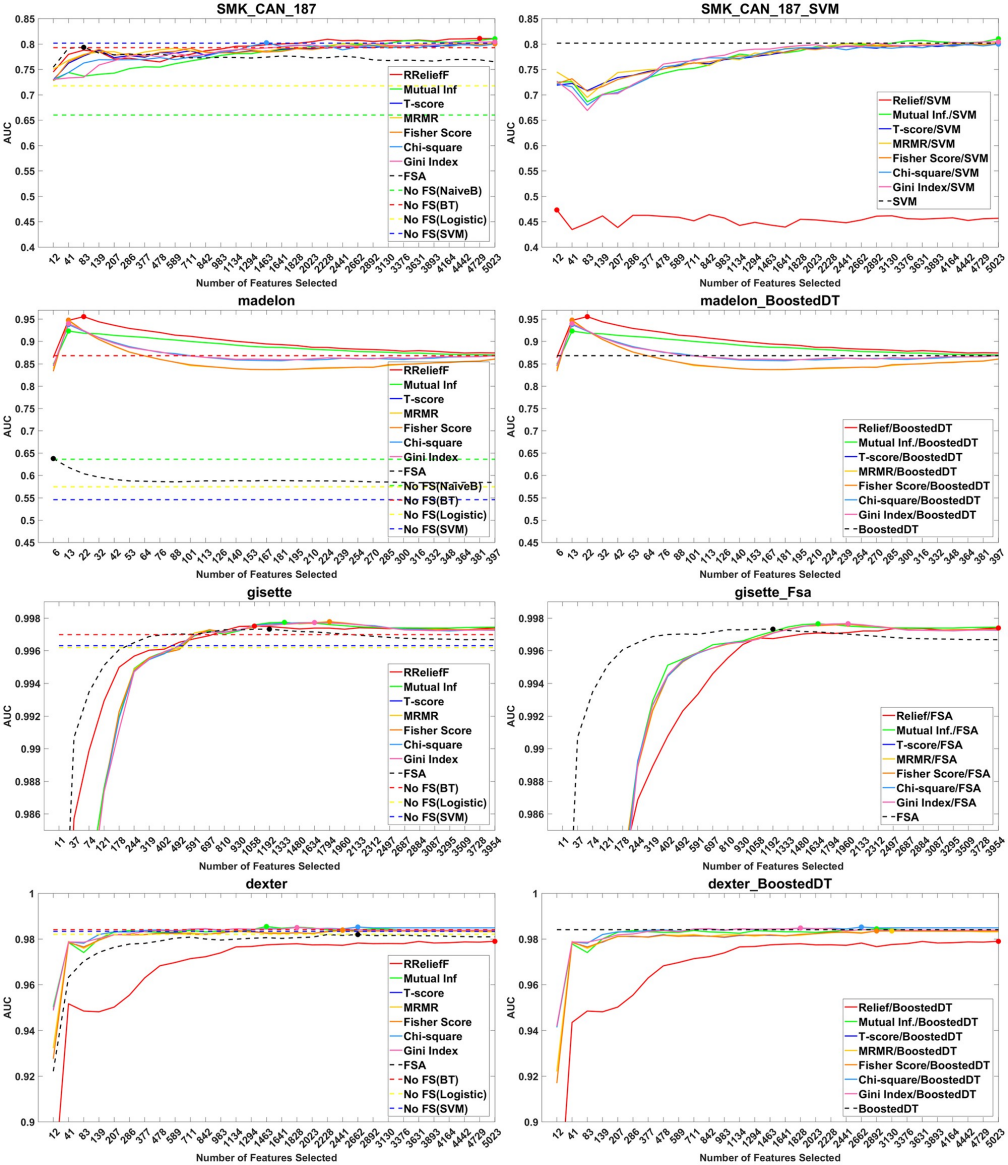
Performance plots of methods with and without feature screening. Left: for each screening method are shown the maximum *R*^2^ value across all learners. Right: *R*^2^ of the screening methods with the best learner for each dataset.

In Fig 3, left are shown the AUC of the best learning algorithm vs. the number of features selected by a screening method for four of the classification datasets. In Fig 3, right, are shown the AUC of the best overall learning algorithm in each case (SVM for SMK_CAN_187, Boosted trees for Madelon and Dexter, FSA for Gisette) vs. the number of features selected by a screening method.

The plots show that all screening methods help obtain better results on the Gisette and Madelon datasets and most screening methods help obtain better results on the SMK_CAN_187 data. It can be observed that on the SMK_CAN_187 and Madelon, although some screening methods show better results, they select a higher number of selected features than FSA when they reach their optimal values. The right side figure of the SMK_CAN_187 plots shows that Relief doesn’t work well with the best learner for this dataset. Only three out of the seven methods help obtain a better result on the Dexter dataset.

**Fig 4:**
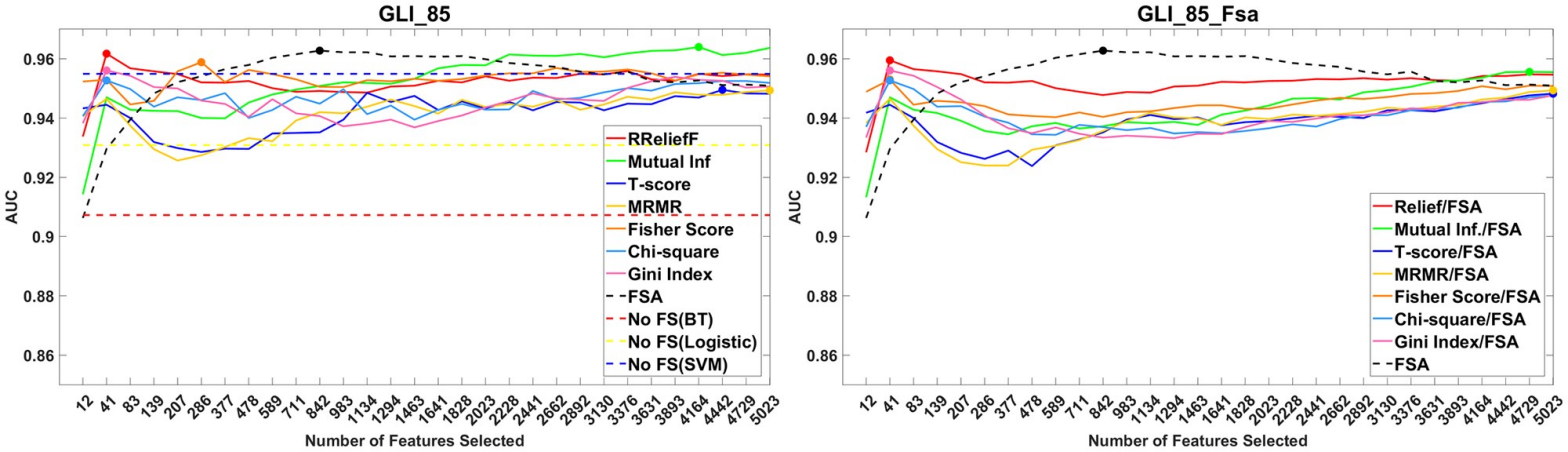
Performance plots of methods with and without feature screening. Left: for each screening method are shown the maximum *R*^2^ value across all learners. Right: *R*^2^ of the screening methods with the best learner for each dataset.

In Fig 4, left are shown the AUC of the best learning algorithm vs. the number of features selected by a screening method on the GLI_85 dataset. In Fig 4, right, are shown the AUC of FSA (for GLI_85) vs. the number of features selected by a screening method. We can observe that only one screening method (mutual information) helps obtain better results than the best learning algorithm without screening.

#### Comparison tables

In Table 6 is shown the number of datasets on which a filter method helps an algorithm perform significantly better, and the number of datasets on which the filter method helps the best performing learning algorithm perform even better. We see that for each learner there is at least one dataset on which a screening method can improve the performance. Mutual information, Relief and Fisher score have best performance among all methods. It is also clear that the screening methods can generally improve the performance of logistic regression and Naive Bayes on 4 to 5 out of the 5 datasets. When compared to best leaner without screening methods, Relief shows to be slightly weaker than the others.

**Table 6:** Overview of the number of datasets where each feature screening method performed significantly better than no screening for different learning algorithms and than the best performing algorithm (larger numbers are better).

Screening Method	Boost Tree	FSA	SVM	NB	Logistic	Best algorithm
Mutual Information	2	1	3	5	5	2
Fisher Score [23]	3	1	2	5	5	2
Chi-square Score [22]	2	1	2	5	4	2
Gini Index [24]	2	1	2	5	4	2
Relief [3]	3	2	1	5	4	1
T-score [1]	2	1	2	5	5	2
MRMR [5]	2	1	2	5	4	2

In Table 7 is shown that Relief only worked on the Madelon dataset. The other screening methods worked on both Gisette and Madelon datasets. It is also shown that only in a few occasions the screening methods harm the best learning algorithm. This is shown by the blank cells in the table. Overall, except Relief, the screening methods have similar performance on the five classification datasets.

**Table 7:** Datasets on which a screening method performs similarly (shown by an “=” sign) or helps perform significantly better (shown by a “*” sign) than the best performing algorithm with no screening, and the total count for the “*” (larger numbers are better).

Screening Method	Dexter	Gisette	*SMK*_*CAN*_187	Madelon	*GLI*_85	Total count
Chi-square Score	=	*	=	*		2
Gini Index	=	*	=	*	=	2
Relief		=	=	*	=	1
Mutual Information	=	*	=	*	=	2
T-score	=	*	=	*		2
Fisher Score	=	*	=	*	=	2
MRMR	=	*	=	*		2

In Table 8 is shown for each screening method-learning algorithm combination the number of datasets for which it was in the top performing group. We can observe that the screening methods with boosted trees and FSA have the overall best performance. Among them, boosted trees and FSA with four screening methods (Chi-square Score, Gini Index, Relief and Mutual Information) have a slight advantage compared to the algorithms without screening. SVM worked well with Mutual Information. The above named four screening methods also helped Logistic regression on one dataset. Naive Bayes didn’t perform well on these five datasets.

**Table 8:** Number of datasets where each combination was in the top performing group.

Learners	Boost Tree	FSA	SVM	NB	Logistic
Filter					
Mutual Information	2	2	2	0	1
Gini Index	2	2	1	0	1
Chi-square Score	2	2	0	0	1
Relief	2	2	0	0	1
T-score	1	1	1	0	0
MRMR	1	1	1	0	0
Fisher Score	1	1	1	0	0
—	1	1	1	0	0

In Table 9, are shown the number of times each screening method was in the top performing group. In the first column, these methods were counted with respect to the learning algorithms they were applied. So there can be at most 25 counts (for each screening method there are five learning algorithms and five datasets) in each cell. The second column shows the counts with the best learning algorithm, so there can be at most 5 counts in each cell. The Mutual Information has the highest counts. It’s significantly higher than no screening. Gini Index, Relief and Chi-square score also have relative higher counts when considering them together with a learning algorithm. Mutual Information and Gini Index have good performance on more datasets than using no screening, when considering only the best learning algorithm for each method and each dataset.

**Table 9:** Ranking of feature screening methods for classification by the number of times each was in the top performing group. (larger numbers are better).

Screening Method	Top performing	
	Method-Algorithm	Method
Mutual Information	7	4
Gini Index	6	4
Chi-square Score	5	3
Relief	5	3
T-score	3	2
MRMR	3	2
Fisher Score	3	2
No Screening	3	3

## Discussion

Since we are interested in evaluating screening methods on real datasets, we don’t have information about the true features that are relevant in connection with the response, so we can only look at prediction performance. In this respect, there are at least two ways to see whether the screening methods are useful for real datasets.

If we ask whether they are helpful in improving the prediction performance of the best learning algorithm from our arsenal, then the answer is “Some of them are sometimes useful, on two datasets out of five, in both regression and classification”. Indeed, for regression we see from Table 3 that Mutual Information and RReliefF were helpful in improving the prediction of the best learning algorithm on two datasets out of five, while the other two screening methods were only helpful on one dataset. For classification, we see from Table 7 that most screening methods were helpful in improving the prediction of the best learning algorithm on two datasets out of five, except Relief, which was only helpful on one dataset.

If however we are interested in using a screening method to reduce the dataset size, then we might ask whether we lose any prediction performance this way. In this case our answer would be “Usually not, for the right screening method, especially in classification”. In Tables 3 and 7 are shown that only in very few occasions do the screening methods harm the performance of best learning algorithm. For regression, we see from Table 5 that RReliefF is the best in this respect, remaining in the top performing group (with the right algorithm and number of selected features) on 4 out of 5 regression datasets. For classification, from Table 9 we see that Mutual Information and Gini index are the best, remaining in the top performing group (with the right algorithm and number of selected features) on 4 out of 5 classification datasets.

If we had to select one screening method that is most successful at both of these tasks, this method would be Mutual Information. We see that it is the only method that is helpful in improving performance in both regression and classification, and stays in the top performing group on most datasets, for both regression and classification.

## Conclusion

Some of the screening methods that were evaluated in this paper bring an improvement in prediction for some datasets, in both regresison and classification. In the classification tasks, the screening methods with boosted trees give the best overall results. All the seven classification screening methods evaluated help improve the performance of learner to a certain degree. The Mutual Information, Gini Index, Chi-square score and Relief work slightly better than the other methods. It also can be seen from the tables that the screening methods work well especially on learning algorithms that give poor results on their own. Compared to classification, there are fewer screening methods for regression problems. Of the three regression screening methods evaluated, RReliefF and Mutual Information work better than correlation, and improve the best learning algorithm performance on two datasets out of five.

## Supporting information

S1 File Review of screening methods and FSA, experimental hyper-parameters and group tables. (PDF)

S1 Tables P-value tables for all datasets. (RAR)
