# Supplementary material for: Are screening methods useful in feature selection? An empirical study
Source: PLoS One. 2019 Sep 11;14(9):e0220842. doi: 10.1371/journal.pone.0220842 (PMC6738588; doi:10.1371/journal.pone.0220842)
Supplement: S1 File — (PDF) [file pone.0220842.s001.pdf]

Supporting information

1 Review of Screening Methods

In this section we give an overview of some of the existing screening (filter) methods for classification and regression, which will be evaluated in our experiments.

1.1 List of Symbols

The following short list of symbols are used throughout the document.

n	the number of observations
p	the number of variables
k	the number of true features
$S = \{(\mathbf{x}_i, y_i) \in \mathbb{R}^p \times \mathbb{R}, i = 1, ..., n\}$	the data space
X	the $n \times p$ data matrix
$X_j, j = 1, ..., p$	the j-th column/feature of X
$\mathbf{x}_i, i = 1, ..., n$	the i-th observation of X
$x_{ij}, i = 1, ..., nj = 1, ..., p$	the j-th column/feature of the i-th observation of X
$\mathbf{y}$	the $n \times 1$ target vector
$y_i, i = 1, ..., n$	the j-th target value

1.2 Screening Methods for Classification

1.2.1 Mutual Information

The mutual information (a.k.a. information gain) method measures the information shared by two variables of interest, in this case, a feature X_j and the class label $\mathbf{y}$. The mutual information between variable A, where $S_A = \{A \in \mathbb{R}\}$ and variable Y, where $S_Y = \{Y \in \mathbb{R}\}$ can be described as:

$$I(A, Y) = \int_{S_A} \int_{S_Y} p(A, Y) \log \frac{p(A, Y)}{p(A)p(Y)} dAdY \tag{1}$$

where $p(A, Y)$ is the joint probability density of A and Y, while $p(A)$ and $p(Y)$ are the marginal p.d.f.s of A and Y.

In practice, given a sample dataset, each feature can be discretized into bins based on the value range. Here, $b = 1, 2, ..., B$ indicates bin number, $c = 1, 2, ..., C$ indicates class number. Therefore mutual information between label vector $\mathbf{y}$ and feature vector X_j can also be described as:

$$I(X_j, \mathbf{y}) = \sum_{b=1}^{B} \sum_{c=1}^{C} p(X_{j_b}, \mathbf{y}_c) \log \frac{p(X_{j_b}, \mathbf{y}_c)}{p(X_{j_b})p(\mathbf{y}_c)} \tag{2}$$

where $p(X_{j_b}, \mathbf{y}_c)$ is the joint probability of bin X_{j_b} and label vector $\mathbf{y}_c$, while $p(X_{j_b})$ and $p(\mathbf{y}_c)$ are the marginal probabilities. Features that are more related to the classification label tend to have higher mutual information.

1.2.2 Relief and ReliefF

The idea of the Relief algorithm is to measure how well a feature's values can distinguish instances that are near each other. For the i-th instance-label pair $(\mathbf{x}_i, y_i)$, denote its nearest instance neighbor from the same class as the nearest hit $(\mathbf{x}_i^{hit}, y_i)$, and its nearest instance neighbor from a different class as the nearest miss $(\mathbf{x}_i^{miss}, y_i^{miss})$. The distance between two instances $\mathbf{x}_i, \mathbf{x}_j$ is calculated using the Euclidean norm $\|\mathbf{x}_i - \mathbf{x}_j\|$. Then the Relief measure for a certain feature F can be computed as:

$$Relief_j = \frac{1}{n}\sum_{i=1}^{n}[\text{diff}(F : x_i, x_i^{miss}) - \text{diff}(F : x_i, x_i^{hit})] \tag{3}$$

where the function $\text{diff}(F : x, y)$ calculates the difference between the values of feature F for two instances. For discrete features $\text{diff}(F : x, y)$ is defined as:

$$\text{diff}(F : x, y) = \begin{cases} 0; & \text{if } x = y \\ 1; & \text{otherwise} \end{cases} \tag{4}$$

and for a continuous feature X_j as:

$$\text{diff}(F : x, y) = \frac{|x - y|}{\max(F) - \min(F)} \tag{5}$$

The Relief measure can also be extended to a multi-class version ReliefF, but we are only interested in binary classification in this paper. In summary, higher Relief values indicate better discrimination power of the label by the feature values.

1.2.3 Minimum Redundancy Maximum Relevance

The minimum redundancy maximum relevance (MRMR) method is set to choose the feature that has the highest mutual information difference (MID) or mutual information quotient (MIQ). The MID and MIQ are calculated as :

$$MID_j = I(X_j, \mathbf{y}) - \frac{1}{|Q|}\sum_{q \in Q} I(X_j, X_q) \tag{6}$$

$$MIQ_j = \frac{I(X_j, \mathbf{y})}{\frac{1}{|Q|}\sum_{q \in Q} I(X_j, X_q)} \tag{7}$$

where Q is the set of features already selected, $I(X_j, \mathbf{y})$ is the mutual information for j-th feature and the label vector $\mathbf{y}$, and $I(X_j, X_q)$ denotes the mutual information between features j and q.

In the case where the features take continuous values, MIQ and MID can be modified as the F-test correlation difference (FCD) and F-test correlation quotient (FCQ). FCD and FCQ are computed as:

$$FCD_j = F(X_j, \mathbf{y}) - \frac{1}{|Q|}\sum_{q \in Q} |c(X_j, X_q)| \tag{8}$$

$$FCQ_j = \frac{F(X_j, \mathbf{y})}{\frac{1}{|Q|}\sum_{q\in Q}|c(X_j, X_q)|} \tag{9}$$

where $F(X_j, \mathbf{y})$ is the F-statistic for j-th feature and label vector $\mathbf{y}$, and $|c(X_j, X_q)|$ denotes the absolute correlation coefficient between features j and q. In the case of binary labels the F-statistic can be replaced by the T-statistic.

1.2.4 T-Score

The T-score method is a feature screening method applied on datasets with binary labels. The method is based on the calculation of the t-statistic. The basic idea is to divide each feature's values into two sample groups based on their labels. Then the t-statistic is calculated to examine if the two sample groups have statistically significant differences in their means. For each feature X_j, the values of X_j are divided into two groups based on their labels. Then the means μ_1 and μ_2 are calculated as the means of the two groups and σ_1 and σ_2 are standard deviations of these two groups respectively. Let n_1 and n_2 be the number of instances of the two groups. Then the t-statistic for feature i can be calculated as:

$$T_j = \frac{|\mu_1 - \mu_2|}{\sqrt{\frac{\sigma_1^2}{n_1} + \frac{\sigma_2^2}{n_2}}} \tag{10}$$

Generally speaking, the higher the t-statistic, the more separated the two labels are by values of that feature and therefore the more relevant that feature is for classification.

1.2.5 Chi-square Score

The chi-square score method is based on the chi-square statistic. It can test the independence between two variables, therefore it can also test the relevance of a variable X_j for the label vector $\mathbf{y}$. If feature X_j has L levels (discretized if necessary) and $\mathbf{y}$ has $C = 2$ levels (label categories), let n_{lc} denote the number of instances with label c and level l for feature j. Let $\hat{n}_{lc}$ denote the estimated number of instances with label c and having level l, $\hat{n}_{lc} = \frac{n_l n_c}{n}$, where n is the total number of instances, n_l is the number of instances having level l, and n_c is the number of instances with label c. The chi-square statistic is then computed as:

$$\chi_j^2 = \sum_{l=1}^{L}\sum_{c=1}^{C}\frac{(n_{lc} - \hat{n}_{lc})^2}{\hat{n}_{lc}} \tag{11}$$

Usually, a higher chi-square statistic indicates low independence, in other word, a higher relevance between that feature and label.

1.2.6 Gini Index

The Gini index method is based on the Gini impurity after splitting a sample set. For a given feature X_j, let $A_h = \{i, x_{ij} \leq h\}$ denote the instances whose values of the $j-$th feature is smaller than or equal to h and $B_h = \{i, x_{ij} > h\}$. The Gini impurity for subset A_h or B_h can be expressed as:

$$Gini(A_h) = 1 - \sum_{c=1}^{C} P(C_c|A_h)^2 \tag{12}$$

where C is the number of labels and $c \in \{1, 2, ..., C\}$ are the label categories. $P(C_c|A_h)$ is the conditional probability of instances having label c given that they are in subset A_h. Let a_c denote the number of instances in A_h with label c. Let a_h denote the number of instances in A_h. Then $P(C_c|A_h)$ can be calculated as a_c/a_h.

Based on these notations, the Gini index after splitting is:

$$Gini_{split} = P(A_h)Gini(A_h) + P(B_h)Gini(B_h) \tag{13}$$

where P(A_h) is the number of instances in subset A_h divided by the number of total instances. Therefore for each feature, the Gini index can be calculated as:

$$Gini_j = P(A_h)(1 - \sum_{c=1}^{C} P(C_c|A_h)^2) + P(B_h)(1 - \sum_{c=1}^{C} P(C_c|B_h)^2) \tag{14}$$

Basically, the Gini index measures the frequency that a randomly chosen instance from the sample set would be incorrectly labeled. So for all possible thresholds h of one feature, select the minimum Gini index as this feature's Gini index. Features with smaller Gini index are preferred.

1.2.7 Fisher Score

The idea of the Fisher score is to choose the feature subset, for which the observations have the largest possible between class distances and the smallest possible within class distances. This would be the feature subset that has the largest Fisher score. The Fisher score for any feature set is computed as:

$$Fisher = Tr(D_b)(D_t + \gamma I)^{-1} \tag{15}$$

where γ is a regularization term, D_b is called between-class scatter matrix, D_t is called total scatter matrix. Since for a certain feature subset with size d, there are $\binom{m}{d}$ combinations of Fisher scores to be calculated, this is too computationally expensive. For this reason, a heuristic is to compute the scores for each feature with respect to the Fisher score criterion. The individual Fisher score is computed as:

$$Fisher_j = \frac{\sum_{c=1}^{C} n_c(\mu_c - \mu)^2}{\sum_{c=1}^{C} n_c \sigma_c^2} \tag{16}$$

where μ and σ are mean and standard deviation of that feature, and μ_c is the mean of the feature values for observations with label c and n_c is the number of instances with label c. Features with larger Fisher scores are preferred.

1.3 Screening Methods for Regression

1.3.1 Correlation

The correlation feature screening method is based on the calculation of correlation coefficient between response and features. It is evaluated as following:

$$\rho_j = \frac{cov(X_j, \mathbf{y})}{\sigma_{\mathbf{y}} \sigma_{X_j}} \tag{17}$$

where X_j is $j-$th feature, $\mathbf{y}$ is response. Features with larger correlation coefficient are preferred.

1.3.2 Mutual Information

To apply mutual information for regression data, we discretize both the feature and the response into a numbers of bins. For feature X_j and response $\mathbf{y}$, let x_{jb} and y_l indicate values falling in b-th and l-th bins respectively. The mutual information for the j-th feature is computed as:

$$I(X_j, Y) = \sum_{b=1}^{B}\sum_{l=1}^{L} P(x_{jb}, y_l) \log \frac{P(x_{jb}, y_l)}{P(x_{jb})P(y_l)} \tag{18}$$

Let n denote the number of instances. Then $P(x_{jb}, y_l)$ can be estimated by N_{jbl}/n, where N_{jkl} is the number of instances falling into feature bin b and response bin l. Also, $P(x_{jb})$ can be estimated by N_{jb}/n, where N_{jb} is the number of instances lay in feature bin b, and $P(y_l)$ can be estimated by N_l/n, where N_l is the number of instances lay in response bin l. Features with larger mutual information have more influence on the response.

1.3.3 RReliefF

RReliefF is a regression version of Relief. It starts from the original weight function. For feature A the function can be expressed as:

$$\begin{aligned} W(A) &= P(\text{different value of A}|\text{nearest instance from different class}) \\ &\quad - P(\text{different value of A}|\text{nearest instance from the same class}) \end{aligned} \tag{19}$$

Denote

$$\begin{aligned} P_{diffA} &= P(\text{different value of A}|\text{nearest instances}) \\ P_{diffP} &= P(\text{different response}|\text{nearest instances}) \\ P_{diffP|diffA} &= P(\text{different response}|\text{different value of A and nearest instances}). \end{aligned} \tag{20}$$

Then from (19), using Bayes' rule:

$$W(A) = \frac{P_{diffP|diffA}P_{diffA}}{P_{diffP}} - \frac{(1 - P_{diffP|diffA})P_{diffA}}{1 - P_{diffP}}, \tag{21}$$

which can be further modified as:

$$W(A) = \frac{N_{dP\&dA}}{N_{dP}} - \frac{(N_{dA} - N_{dP\&dA})}{m - N_{dP}} \tag{22}$$

where N_{dA}, N_{dP} and $N_{dP\&dA}$ denote different feature value, different response value and different feature & response value respectively. Denote for instance $\mathbf{x}_i$ its k-nearest instances as $\mathbf{u}_{ij}, j \in \{1, ..., k\}$. Then the expressions for N_{dA}, N_{dP} and $N_{dP\&dA}$ are:

$$\begin{aligned} N_{dA} &= \sum_{i=1}^{n}\sum_{j=1}^{k} \text{diff}(A : \mathbf{x}_i, \mathbf{u}_{ij}) d(i, j) \\ N_{dP} &= \sum_{i=1}^{n}\sum_{j=1}^{k} \text{diff}(y : \mathbf{x}_i, \mathbf{u}_{ij}) d(i, j) \\ N_{dP\&dA} &= \sum_{i=1}^{n}\sum_{j=1}^{k} \text{diff}(y : \mathbf{x}_i, \mathbf{u}_{ij})\, \text{diff}(A : \mathbf{x}_i, \mathbf{u}_{ij}) d(i, j) \end{aligned} \tag{23}$$

Where $\text{diff}(F, x, y)$ is defined in Eq. (4) and (5) and $d(i, j)$ is used to take account the distance between $\mathbf{x}_i$ and $\mathbf{u}_j$:

$$d(i, j) = \frac{d_1(i, j)}{\sum_{l=1}^{k} d_1(i, l)} \tag{24}$$

and

$$d_1(i, j) = \exp(-\text{rank}^2(\mathbf{x}_i, \mathbf{u}_{ij})/\sigma^2) \tag{25}$$

where $\text{rank}(\mathbf{x}_i, \mathbf{u}_{ij})$ is the rank of the instance $\mathbf{u}_{ij}$ in a sequence of instances ordered by the distance from $\mathbf{x}_i$, and σ is a user defined parameter. $d_1(i, j)$ is calculated in an exponentially decreasing fashion with the idea that further instances should have lesser influence. Usually, $d_1(i, j)$ takes value $1/k$. Features with larger $W(\cdot)$ are preferred.

1.4 Feature Selection With Annealing (FSA)

Feature Selection With Annealing (a.k.a. FSA) is a recent embedded method for feature selection that can handle high dimensional data. FSA can bring the relevant feature space down to an acceptable level using an variable removal schedule and obtain a rather accurate and stable model. The basic algorithm of FSA is:

Algorithm 1 Feature Selection with Annealing (FSA)

Input: Training samples $(\mathbf{x}_i, y_i) \in \mathbb{R}^p \times \mathbb{R}, i = 1, 2, ..., N$.
Output: Trained model parameter vector $\boldsymbol{\beta}$.

1: Initialize $\boldsymbol{\beta}$.
2: **for** e=1 to N^{iter} **do**
3: Update $\boldsymbol{\beta} \leftarrow \boldsymbol{\beta} - \eta \frac{\partial L(\boldsymbol{\beta})}{\partial \boldsymbol{\beta}}$
4: Keep the M_e features with highest $|\boldsymbol{\beta}_j|$ and renumber them 1, ..., M_e.
5: **end for**

The value of N^{iter} in step 2 is the total number of iterations. The formula in step 3 uses a typical gradient descent or an epoch of stochastic gradient descent with momentum and minibatch towards minimizing the loss $L(\boldsymbol{\beta})$. The M_e in step 4 is the annealing schedule which gradually decreases with the iteration number e. It decides how many features to keep in each iteration. Let k be a user defined parameter controlling how many features to keep in the end. The M_e can be computed as:

$$M_e = k + (p - k)\max(0, \frac{N^{iter} - 2e}{2e\mu + N^{iter}}), e = 1, ..., N^{iter} \tag{26}$$

where p is the feature dimension of the original input data and μ is the annealing parameter which can be tuned using cross validation. FSA has good computational efficiency and theoretical guarantees of consistency. The user defined parameter k denoting how many features to select is more intuitive than the penalty parameter in the penalized methods (e.g. L_1 penalized regression) and makes the procedure more controllable.

2 Learning algorithm hyper-parameters

Some learning algorithms such as FSA and boosted trees have their performance highly dependent on the values of the hyper-parameters. To avoid any confounding effect of the method for selecting these parameters (e.g. by cross-validation or AIC/BIC), these learning algorithms were run on a discrete set of combinations on a single

training/validation split of the data, and the parameter combination that obtained the best validation result was used in the entire experiment. The values that were used are given in Tables 1 and 2. The other learning algorithms were built-in Matlab and we used the default values for all parameters.

Table 1. Selected parameter values for FSA.

Parameters	BMI	Tumor	CoEPrA2006	Indoorloc	Wikiface
learning rate η	0.00001	0.000003	0.0001	0.00001	0.00005
number of epochs N^{iter}	150	50	100	250	450
annealing parameter μ	800	30	650	200	250
minibatch size	285	250	15	30	150
shrinkage parameter	0.0001	0.001	0.9	0.0001	0.001
Parameters	Gisette	Dexter	Madelon	SMK_CAN_187	GLI_85
learning rate η	0.0001	0.000001	0.0005	0.01	0.1
number of epochs N^{iter}	60	300	10	500	800
annealing parameter μ	600	100	40	280	100
minibatch size	20	30	145	145	100
shrinkage parameter	0	0	0.00001	0.001	0.005

Table 2. Selected parameter values for boosted trees.

Parameters	BMI	Tumor	CoEPrA2006	Indoorloc	Wikiface
max number of splits	1	1	1	8	1
boosting iterations	100	10	10	500	400
Parameters	Gisette	Dexter	Madelon	SMK_CAN_187	GLI_85
max number of splits	4	4	2^6	1	2
boosting iterations	400	400	1900	500	200

3 Table of groups

In this section we present the summary of the performance of each screening method-learning algorithm combination and their division into groups such that the difference between the best method and the worst method in each group is not significant at the 0.05 level.

In Table 3 are shown the groups, the mean R^2 of test data and standard error of mean estimation obtained over all the runs for the BMI dataset. Also shown are the number of features ω selected by the screening method and the number of features κ selected by the learning algorithm where the average R^2 is maximum. From Table 3 we see that the best learner is FSA and that the FSA results with and without screening methods belong to the same group indicating that the screening methods don't improve the performance of FSA significantly. For ridge regression, the performance of RReliefF and Mutual information belongs to a group higher than ridge regression without screening. For boosted regression trees, the screening methods do provide a significant improvement. We can also see that the number of features selected by FSA is smaller than the number of features selected by the screening methods. So for FSA, the features selected by screening methods can still be reduced in order to get the best result.

The same types of results are shown in Table 4 for the tumor dataset. Again, the best results are obtained with FSA and the FSA results without screening methods belong to the first group. So screening methods do not improve the performance of FSA in this case either. For ridge regression and boosted regression trees, the results with screening methods belong to higher tier groups than results without screening method, which means the screening methods help those two learners. Also for FSA, the number

Table 3. Table of groups, BMI dataset. SE is the standard error of mean estimation, ω is the number of features selected by the screening method, κ is the number of features selected by FSA.

Group		Screening Methods	Learner	Mean	SE	ω	κ
A		RReliefF	FSA	0.7632	0.0006	1758	692
A		—	FSA	0.7607	0.0005	—	839
A		Mutual Information	FSA	0.7606	0.0006	3537	1550
A		Correlation	FSA	0.7590	0.0006	5856	1354
B		Correlation	Ridge	0.7238	0.0008	5140	—
B	C	RReliefF	Ridge	0.7172	0.0005	6230	—
D	C	Mutual Information	Ridge	0.7078	0.0009	6230	—
D		—	Ridge	0.7073	0.0004	—	—
E		Mutual Information	Boosted Reg. Trees	0.5198	0.0020	13	—
E		RReliefF	Boosted Reg. Trees	0.5157	0.0027	13	—
E		Correlation	Boosted Reg. Trees	0.4932	0.0018	13	—
F		—	Boosted Reg. Trees	0.2520	0.0043	—	—

Table 4. Table of groups, Tumor dataset. SE is the standard error of mean estimation, ω is the number of features selected by the screening method, κ is the number of features selected by FSA.

Group		Screening Methods	Learner	Mean	SE	ω	κ
A		—	FSA	0.3473	0.0001	—	558
A	B	RReliefF	FSA	0.3472	0.0001	6230	2210
C	B	Correlation	FSA	0.3427	0.0001	6230	1550
C		Mutual Information	FSA	0.3404	0.0001	6230	1758
D		Mutual Information	Ridge	0.2949	0.0001	13	—
D		Correlation	Ridge	0.2925	< 0.0001	13	—
D	E	Correlation	Boosted Reg. Trees	0.2855	0.0004	13	—
D	E	Mutual Information	Boosted Reg. Trees	0.2840	0.0005	13	—
	E	RReliefF	Ridge	0.2831	0.0001	13	—
	E	RReliefF	Boosted Reg. Trees	0.2738	0.0003	93	—
F		—	Boosted Reg. Trees	0.2272	0.0003	—	—
F		—	Ridge	0.2153	0.0003	—	—

features selected by screening methods is further reduced in order to get the maximum result.

In Table 5 are shown the results for the CoEPrA2006_3 dataset. Again the FSA without screening is in the top group. The results with screening methods for Ridge regression belong to higher tier groups than without screening. The results of boosted regression trees with or without screening belong to the same group. So in this case, the screening methods only improve the performance of ridge regression.

In Table 6 are shown the results for the Indoorloc dataset. Here we see that two results with screening methods for Boosted Trees belong to higher tier group than without screening. There are no screening methods that give higher tier results than no screening for FSA and ridge regression.

In Table 7 are shown the results for the Wikiface dataset. All screening methods applied to ridge regression belong to higher tier groups than ridge regression without screening, whereas only the correlation method on boosted trees shows improvement for the other two learners.

In Table 8 are shown the results for Dexter, a classification dataset. We see that for

Table 5. Table of groups, CoEPrA2006_3 dataset. SE is the standard error of mean estimation, ω is the number of features selected by the screening method, κ is the number of features selected by FSA.

Group			Screening Methods	Learner	Mean	SE	ω	κ
A			Correlation	Ridge	0.2858	0.0046	208	—
A	B		—	FSA	0.2844	0.0049	—	65
A	B		RReliefF	FSA	0.2815	0.0061	2940	65
A	B		Correlation	FSA	0.2747	0.0044	971	412
C	B		RReliefF	Ridge	0.2482	0.0052	971	—
C			Mutual Information	FSA	0.2227	0.0049	3112	65
D			Mutual Information	Ridge	0.0763	0.0053	412	—
D	E		RReliefF	Boosted Reg. Trees	0.0746	0.0064	491	—
D	E	F	Correlation	Boosted Reg. Trees	0.0661	0.0064	668	—
D	E	F	—	Boosted Reg. Trees	0.0362	0.0049	—	—
	E	F	Mutual Information	Boosted Reg. Trees	0.0082	0.0019	65	—
		F	—	Ridge	0	0	—	—

Table 6. Table of groups, Indoorloc dataset. SE is the standard error of mean estimation, ω is the number of features selected by the screening method, κ is the number of features selected by FSA.

Group	Screening Methods	Learner	Mean	SE	ω	κ
A	Mutual Information	Boosted Reg. Trees	0.9703	< 0.0001	254	—
A	RReliefF	Boosted Reg. Trees	0.9698	< 0.0001	285	—
B	—	Boosted Reg. Trees	0.9685	< 0.0001	—	—
B	Correlation	Boosted Reg. Trees	0.9681	< 0.0001	381	—
C	Mutual Information	Ridge	0.9198	< 0.0001	397	—
C	—	Ridge	0.9198	< 0.0001	—	—
D	Correlation	Ridge	0.9188	< 0.0001	397	—
E	—	FSA	0.9182	< 0.0001	—	397
F	Mutual Information	FSA	0.9177	< 0.0001	397	285
G	Correlation	FSA	0.9167	< 0.0001	397	300
H	RReliefF	Ridge	0.9158	< 0.0001	397	—
I	RReliefF	FSA	0.9139	< 0.0001	397	300

SVM, FSA and boosted trees the results of the learners with screening belong to either the same group or lower groups than learners without screening. Most of the screening methods did a great job in improving the performance of Logistic Regression for this dataset, and all methods improved the performance Naive Bayes. The Relief method didn't work on this data as all of the Relief based combinations are ranked at the end of table. For some of the FSA combinations, the number of selected features by screening methods and number of selected features by FSA are the same, meaning the screening methods already selected the features that can give the best result.

In Table 9 are shown the results for Gisette. Clearly screening methods work on boosted trees by giving results that belong to higher tier groups than the learner alone. Naive Bayes and logistic regression have a similar conclusion as boosted trees. Beside the Relief-FSA combination, the other screening methods applied to FSA and SVM show improvement. For some of the FSA combinations, the number of selected features by screening methods and number of selected features by FSA are the same, meaning the screening methods already selected the features that can give the best result.

In Table 10 are shown the results for the SMK_CAN_187 dataset. The results with

Table 7. Table of groups, Wikiface dataset. SE is the standard error of mean estimation, ω is the number of features selected by the screening method, κ is the number of features selected by FSA.

Group	Screening Methods	Learner	Mean	SE	ω	κ
A	Mutual Information	Ridge	0.3490	< 0.0001	1739	—
B	RReliefF	Ridge	0.3482	< 0.0001	1739	—
C	Correlation	Ridge	0.3478	< 0.0001	1739	—
D	—	Ridge	0.3468	< 0.0001	—	—
E	Mutual Information	FSA	0.3426	< 0.0001	1739	370
E	—	FSA	0.3424	< 0.0001	—	440
E	RReliefF	FSA	0.3424	< 0.0001	1739	440
F	Correlation	FSA	0.3419	< 0.0001	1381	370
F	Correlation	Boosted Reg. Trees	0.2981	< 0.0001	31	—
G	RReliefF	Boosted Reg. Trees	0.2546	< 0.0001	10	—
G	Mutual Information	Boosted Reg. Trees	0.2517	0.0003	955	—
G	—	Boosted Reg. Trees	0.2156	0.0003	—	—

screening for Naive Bayes and Logistic Regression belong to higher tier groups than those without screening. For the other learning algorithms, screening methods give results belonging to the same group or lower groups as learners without screening. This indicates no improvement from using screening for those learners.

In Table 11 are shown the results for Madelon. The results with screening for Naive Bayes, SVM, Boosted Decision Trees and Logistic Regression belong to higher tier groups than those without screening. For FSA, only the result of Relief/FSA belongs to higher tier group than FSA without screening.

In Table 12 are shown the results for the GLI_85 dataset. The results with screening belong to the same group or lower groups than the learner alone for FSA. SVM, Logistic Regression and boosted trees each have a few screening methods that give higher tier results. All screening methods give results belonging to higher groups than learner without screening for Naive Bayes.

Table 8. Table of groups, Dexter dataset. SE is the standard error of mean estimation, ω is the number of features selected by the screening method, κ is the number of features selected by FSA.

Group						Screening Methods	Learner	Mean	SE	ω	κ
A						Mutual Information	Logistic Reg.	0.9854	< 0.0001	1463	—
A						Chi-square Score	Boosted Decision Trees	0.9852	< 0.0001	2662	—
A	B					Chi-square Score	Logistic Reg.	0.9851	< 0.0001	1828	—
A	B	C				Gini Index	Logistic Reg.	0.9850	< 0.0001	1828	—
A	B	C	D			Gini Index	Boosted Decision Trees	0.9848	< 0.0001	1828	—
A	B	C	D			Mutual Information	Boosted Decision Trees	0.9844	< 0.0001	2892	—
A	B	C	D			—	Boosted Decision Trees	0.9841	< 0.0001	—	—
E	B	C	D			Fisher Score	Logistic Reg.	0.9839	< 0.0001	2441	—
E	B	C	D	F		Mutual Information	SVM	0.9838	< 0.0001	3893	—
E		C	D	F		T-score	Logistic Reg.	0.9838	< 0.0001	2441	—
E		C	D	F		MRMR	Logistic Reg.	0.9837	< 0.0001	2441	—
E		C	D	F		MRMR	SVM	0.9835	< 0.0001	4164	—
E	G	C	D	F		T-score	Boosted Decision Trees	0.9835	< 0.0001	2892	—
E	G	C	D	F		Fisher Score	Boosted Decision Trees	0.9835	< 0.0001	2892	—
E	G	C	D	F	H	MRMR	Boosted Decision Trees	0.9835	< 0.0001	3130	—
E	G		D	F	H	—	SVM	0.9834	< 0.0001	—	—
E	G		D	F	H	T-score	SVM	0.9831	< 0.0001	5023	—
E	G	I		F	H	Mutual Information	FSA	0.9830	< 0.0001	1828	1828
	G	I	J	F	H	Chi-square Score	FSA	0.9827	< 0.0001	1828	1828
	G	I	J	F	H	Gini Index	FSA	0.9827	< 0.0001	1828	1828
	G	I	J		H	T-score	FSA	0.9824	< 0.0001	5023	5023
		I	J		H	MRMR	FSA	0.9824	< 0.0001	2662	2441
		I	J		H	Fisher Score	FSA	0.9823	< 0.0001	2662	2441
		I	J		H	Chi-square Score	SVM	0.9822	< 0.0001	3893	—
		I	J		H	Gini Index	SVM	0.9822	< 0.0001	3893	—
		I	J		H	—	Logistic Reg.	0.9820	< 0.0001	—	—
		I	J			—	FSA	0.9819	< 0.0001	—	2662
K			J			Fisher Score	SVM	0.9809	< 0.0001	3130	—
K	L					Relief	Boosted Decision Trees	0.9790	< 0.0001	5023	—
	L					Relief	FSA	0.9780	< 0.0001	3130	377
	L					Relief	Logistic Reg.	0.9761	0.0001	1463	—
M						Mutual Information	Naive Bayes	0.9157	0.0004	83	—
N						MRMR	Naive Bayes	0.9005	0.0002	83	—
N						Chi-square Score	Naive Bayes	0.9002	0.0003	41	—
N						Gini Index	Naive Bayes	0.9002	0.0003	41	—
N						T-score	Naive Bayes	0.8993	0.0001	83	—
N						Fisher Score	Naive Bayes	0.8991	0.0002	83	—
O						Relief	Naive Bayes	0.8005	0.0004	12	—
P						Relief	SVM	0.6628	0.0014	41	—
P						—	Naive Bayes	0.6520	0.0006	—	—

Table 9. Table of groups, Gisette dataset. SE is the standard error of mean estimation, ω is the number of features selected by the screening method, κ is the number of features selected by FSA.

Group			Screening Methods	Learner	Mean	SE	ω	κ
A			MRMR	Boosted Decision Trees	0.9978	< 0.0001	2133	—
A			T-score	Boosted Decision Trees	0.9978	< 0.0001	1634	—
A			Fisher Score	Boosted Decision Trees	0.9978	< 0.0001	1634	—
A			Mutual Information	Boosted Decision Trees	0.9977	< 0.0001	1333	—
A			Gini Index	Boosted Decision Trees	0.9977	< 0.0001	2884	—
A	B		Chi-square Score	Boosted Decision Trees	0.9977	< 0.0001	2312	—
A	B		Chi-square Score	FSA	0.9977	< 0.0001	1634	1634
A	B		Gini Index	FSA	0.9977	< 0.0001	1960	1960
A	B		Mutual Information	FSA	0.9976	< 0.0001	1480	1480
A	B		T-score	FSA	0.9976	< 0.0001	1794	1794
A	B		Fisher Score	FSA	0.9976	< 0.0001	1960	1960
A	B		MRMR	FSA	0.9976	< 0.0001	3954	1058
C	B		Relief	Boosted Decision Trees	0.9975	< 0.0001	1058	—
C			Relief	FSA	0.9974	< 0.0001	2687	1480
C	D		—	FSA	0.9973	< 0.0001	—	1058
E	D		T-score	SVM	0.9973	< 0.0001	1480	—
E	D		Fisher Score	SVM	0.9973	< 0.0001	1634	—
E			Gini Index	SVM	0.9972	< 0.0001	1634	—
E			MRMR	SVM	0.9972	< 0.0001	1960	—
E	F		Chi-square Score	SVM	0.9972	< 0.0001	1634	—
E	F	G	Mutual Information	SVM	0.9971	< 0.0001	1480	—
H	F	G	Mutual Information	Logistic Reg.	0.9970	< 0.0001	2133	—
H	F	G	—	Boosted Decision Trees	0.9970	< 0.0001	—	—
H	F	G	Fisher Score	Logistic Reg.	0.9969	< 0.0001	1960	—
H		G	Chi-square Score	Logistic Reg.	0.9969	< 0.0001	1794	—
H			Gini Index	Logistic Reg.	0.9969	< 0.0001	1960	—
H	I		MRMR	Logistic Reg.	0.9968	< 0.0001	1794	—
H	I		T-score	Logistic Reg.	0.9968	< 0.0001	1794	—
	I		Relief	Logistic Reg.	0.9967	< 0.0001	2497	—
J			—	SVM	0.9963	< 0.0001	—	—
K			—	Logistic Reg.	0.9962	< 0.0001	—	—
L			Mutual Information	Naive Bayes	0.9583	< 0.0001	2312	—
L			MRMR	Naive Bayes	0.9582	< 0.0001	178	—
L			T-score	Naive Bayes	0.9582	< 0.0001	1634	—
L			Fisher Score	Naive Bayes	0.9582	< 0.0001	2687	—
L			Gini Index	Naive Bayes	0.9579	< 0.0001	1794	—
L			Chi-square Score	Naive Bayes	0.9579	< 0.0001	1794	—
M			Relief	Naive Bayes	0.9474	< 0.0001	2687	—
N			—	Naive Bayes	0.9326	< 0.0001	—	—
O			Relief	SVM	0.8914	< 0.0001	1333	—

Table 10. Table of groups, SMK_CAN_187 dataset. SE is the standard error of mean estimation, ω is the number of features selected by the screening method, κ is the number of features selected by FSA.

Group						Screening Methods	Learner	Mean	SE	ω	κ
A						Relief	FSA	0.8107	0.0008	4729	139
A						Mutual Information	SVM	0.81013	0.0009	5023	—
A	B					Mutual Information	FSA	0.8072	0.0013	4729	207
A	B					Gini Index	SVM	0.8039	0.0009	5023	—
A	B					MRMR	SVM	0.8032	0.0006	5023	—
A	B	C				Chi-square Score	FSA	0.8023	0.0010	1463	83
A	B	C	D			Relief	Boosted Decision Trees	0.8019	0.0008	5023	—
A	B	C	D	E		—	SVM	0.8015	0.0009	—	—
A	B	C	D	E		T-score	SVM	0.8012	0.0010	5023	—
	B	C	D	E		Fisher Score	SVM	0.8002	0.0008	5023	—
	B	C	D	E		Chi-square Score	SVM	0.7996	0.0016	5023	—
F	B	C	D	E		Gini Index	FSA	0.7991	0.0018	4442	83
F	B	C	D	E		T-score	Boosted Decision Trees	0.7950	0.0006	2441	—
F	B	C	D	E		MRMR	Boosted Decision Trees	0.7940	0.0010	4442	—
F	B	C	D	E	G	—	FSA	0.7932	0.0013	—	83
F	B	C	D	E	G	—	Boosted Decision Trees	0.7926	0.0007	—	—
F		C	D	E	G	Fisher Score	Boosted Decision Trees	0.7912	0.0012	711	—
F			D	E	G	Gini Index	Boosted Decision Trees	0.7899	0.0010	2023	—
F				E	G	Fisher Score	FSA	0.7895	0.0012	139	12
F				E	G	T-score	FSA	0.7878	0.0009	139	12
F	H				G	MRMR	FSA	0.7871	0.0014	3130	139
F	H				G	Mutual Information	Boosted Decision Trees	0.7818	0.0013	4442	–
F	H				G	Chi-square Score	Boosted Decision Trees	0.7812	0.0008	5023	—
F	H				G	Relief	Logistic Reg.	0.7804	0.0013	2023	—
	H	I			G	Gini Index	Logistic Reg.	0.7706	0.0010	377	—
	H	I				Chi-square Score	Logistic Reg.	0.7689	0.0013	286	—
		I				Fisher Score	Logistic Reg.	0.7646	0.0012	589	—
		I				T-score	Logistic Reg.	0.7642	0.0018	842	—
		I				MRMR	Logistic Reg.	0.7628	0.0011	711	—
		I				Mutual Information	Logistic Reg.	0.7567	0.0017	589	—
J						MRMR	Naive Bayes	0.7409	0.0010	12	—
J	K					Fisher Score	Naive Bayes	0.7312	0.0008	41	—
	K	L				T-score	Naive Bayes	0.7291	0.0008	41	—
	K	L	M			Chi-square Score	Naive Bayes	0.7279	0.0009	41	—
	K	L	M			Gini Index	Naive Bayes	0.7249	0.0005	41	—
		L	M			Mutual Information	Naive Bayes	0.7238	0.0008	41	—
		L	M			—	Logistic Reg.	0.7174	0.0015	—	—
			M			Relief	Naive Bayes	0.7133	0.0009	12	—
N						—	Naive Bayes	0.6599	0.0005	—	—
O						Relief	SVM	0.4730	0.0006	12	—

Table 11. Table of groups, Madelon dataset. SE is the standard error of mean estimation, ω is the number of features selected by the screening method, κ is the number of features selected by FSA.

Group			Screening Methods	Learner	Mean	SE	ω	κ
A			Relief	Boosted Decision Trees	0.9554	< 0.0001	22	—
B			T-score	Boosted Decision Trees	0.9476	< 0.0001	13	—
B			Fisher Score	Boosted Decision Trees	0.9476	< 0.0001	13	—
C			MRMR	Boosted Decision Trees	0.9460	< 0.0001	13	—
D			Gini Index	Boosted Decision Trees	0.9398	< 0.0001	13	—
E			Chi-square Score	Boosted Decision Trees	0.9376	< 0.0001	13	—
F			Mutual Information	Boosted Decision Trees	0.9232	< 0.0001	13	—
G			—	Boosted Decision Trees	0.8679	< 0.0001	—	—
H			Relief	Naive Bayes	0.6884	< 0.0001	13	—
I			T-score	Naive Bayes	0.6832	< 0.0001	13	—
I			Fisher Score	Naive Bayes	0.6832	< 0.0001	13	—
I	J		Gini Index	Naive Bayes	0.6821	< 0.0001	22	—
	J		Mutual Information	Naive Bayes	0.6818	< 0.0001	13	—
	J		MRMR	Naive Bayes	0.6817	< 0.0001 22	—	
	J		Chi-square Score	Naive Bayes	0.6815	< 0.0001	22	—
K			Relief	FSA	0.6394	< 0.0001	42	6
K	L		Relief	Logistic Reg.	0.6389	< 0.0001	6	—
K	L		Mutual Information	SVM	0.6386	< 0.0001	6	—
K	L	M	MRMR	FSA	0.6384	< 0.0001	364	6
K	L	M	T-score	FSA	0.6384	< 0.0001	364	6
K	L	M	Fisher Score	FSA	0.6384	< 0.0001	364	6
K	L	M	Chi-square Score	FSA	0.6384	< 0.0001	364	6
K	L	M	Gini Index	FSA	0.6381	< 0.0001	364	6
	L	M	Mutual Information	FSA	0.6381	< 0.0001	42	6
	L	M	T-score	SVM	0.6381	< 0.0001	6	—
	L	M	Fisher Score	SVM	0.6381	< 0.0001	6	—
	L	M	Chi-square Score	SVM	0.6380	< 0.0001	6	—
		M	Mutual Information	Logistic Reg.	0.6379	< 0.0001	6	—
		M	Gini Index	SVM	0.6378	< 0.0001	6	—
		M	—	FSA	0.6377	< 0.0001	—	6
		M	MRMR	SVM	0.6376	< 0.0001	6	—
		M	T-score	Logistic Reg.	0.6373	< 0.0001	6	—
		M	Fisher Score	Logistic Reg.	0.6373	< 0.0001	6	—
		M	Chi-square Score	Logistic Reg.	0.6372	< 0.0001	6	—
		M	Gini Index	Logistic Reg.	0.6368	< 0.0001	6	—
		M	MRMR	Logistic Reg.	0.6366	< 0.0001	6	—
		M	—	Naive Bayes	0.6360	< 0.0001	—	—
N			Relief	SVM	0.6120	0.0001	6	—
O			—	Logistic Reg.	0.5744	0.0002	—	—
P			—	SVM	0.5455	0.0002	—	—

Table 12. Table of groups, GLI_85 dataset. SE is the standard error of mean estimation, ω is the number of features selected by the screening method, κ is the number of features selected by FSA.

Group				Screening Methods	Learner	Mean	SE	ω	κ
A				Mutual Information	SVM	0.9639	0.0014	4164	—
A				—	FSA	0.9627	0.0006	—	842
A				Relief	Logistic Reg.	0.9616	0.0009	41	—
A	B			Relief	FSA	0.9594	0.0010	41	41
A	B	C		Fisher Score	SVM	0.9587	0.0010	286	—
A	B	C	D	Gini Index	FSA	0.9560	0.0014	41	12
	B	C	D	Mutual Information	FSA	0.9555	0.0005	4729	711
	B	C	D	—	SVM	0.9548	0.0007	—	—
	B	C	D	Gini Index	SVM	0.9538	0.0011	3893	—
E		C	D	Fisher Score	FSA	0.9529	0.0012	41	12
E		C	D	Chi-square Score	FSA	0.9526	0.0011	41	12
E		C	D	Chi-square Score	SVM	0.9524	0.0009	4442	—
E	F	C	D	Mutual Information	Logistic Reg.	0.9519	0.0016	4729	—
E	F	C	D	Fisher Score	Logistic Reg.	0.9516	0.0008	2892	—
E	F		D	T-score	SVM	0.9494	0.0008	4442	—
E	F		D	MRMR	FSA	0.9492	0.0008	5023	711
E	F		D	MRMR	SVM	0.9486	0.0011	3893	—
E	F		D	T-score	FSA	0.9482	0.0007	5023	711
E	F		D	T-score	Logistic Reg.	0.9478	0.0008	4729	—
E	F	G	D	Chi-square Score	Logistic Reg.	0.9450	0.0017	4729	—
E	F	G		Gini Index	Logistic Reg.	0.9449	0.0009	2662	—
	F	G		MRMR	Logistic Reg.	0.9445	0.0006	3893	—
H	F	G		Relief	Boosted Decision Trees	0.9395	0.0027	139	—
H	F	G		Relief	Naive Bayes	0.9393	0.0007	41	—
H	F	G		Relief	SVM	0.9379	0.0013	139	—
H		G		Fisher Score	Boosted Decision Trees	0.9357	0.0012	41	—
H	I	G		—	Logistic Reg.	0.9308	0.0023	—	—
H	I	G		Mutual Information	Boosted Decision Trees	0.9285	0.0016	83	—
H	I	G		T-score	Boosted Decision Trees	0.9265	0.0024	12	—
H	I	G		Gini Index	Boosted Decision Trees	0.9249	0.0019	41	—
H	I			MRMR	Boosted Decision Trees	0.9243	0.0016	12	—
H	I			Fisher Score	Naive Bayes	0.9235	0.0015	12	—
H	I			Chi-square Score	Boosted Decision Trees	0.9204	0.0024	41	—
H	I	J		Mutual Information	Naive Bayes	0.9103	0.0012	41	—
	I	J		—	Boosted Decision Trees	0.9072	0.0020	—	—
K		J		MRMR	Naive Bayes	0.8940	0.0011	83	—
K		J		T-score	Naive Bayes	0.8936	0.0013	83	—
K				Chi-square Score	Naive Bayes	0.8833	0.0013	41	—
K				Gini Index	Naive Bayes	0.8821	0.0014	41	—
L				—	Naive Bayes	0.7006	0.0033	—	—
